# Editorial: Promoting inclusion and representation: the role of cultural diversity in sports

**DOI:** 10.3389/fpsyg.2025.1695684

**Published:** 2025-09-19

**Authors:** Mário Borges, António Rosado, Rita F. de Oliveira

**Affiliations:** ^1^School of Applied Sciences, London South Bank University, London, United Kingdom; ^2^Faculty of Human Kinetics, University of Lisbon, Lisbon, Portugal

**Keywords:** cultural diversity, inclusion, representation, social justice, sport equity

As global mobility increases and sport becomes an ever more international arena (Borges et al., 2015), the need to understand and promote inclusion in sports has grown in significance (Corvino et al., 2023). This Research Topic, *Promoting Inclusion and Representation: The Role of Cultural Diversity in Sport*, brings together diverse scholarly perspectives that interrogate the intersections of cultural diversity within the sporting context.

The articles in this Research Topic explore the complex interplay between inclusion, identity, social perceptions, and performance across a range of sporting settings. A central concern is the persistent underrepresentation of ethnic and cultural minorities in sport, particularly in coaching roles (Cunningham, 2020; Cunningham et al., 2021). Drawing on insights from psychology, sociology, education, policy, and media studies, these nineteen articles address both the evolving challenges and the potential benefits of cultural diversity in sport. The Research Topic advances previous recommendations for embedding inclusion across all levels of sport (Cooper et al., 2020; Spaaij et al., 2023). Authored by seventy-six researchers, from fifteen countries, it includes two systematic reviews, two brief reports and fifteen original research articles, offering a diverse array of academic contributions.

Persistent underrepresentation of minoritised groups in coaching and leadership roles is explored in several studies. Marcelino et al. examine the barriers faced by Black coaches in Brazilian basketball, identifying racism, lack of visibility, and accumulated prejudice. Complementing this, Malcomb and Zitek critique Major League Baseball's diversity metrics which may obscure the stagnation and exclusion faced by international Latino coaches and players. Malcomb et al. further explore intercultural challenges in Minor League Baseball, emphasizing language barriers and institutional neglect. Dias et al. highlight gender disparities in youth coaching across 24 countries, particularly the neglect of female biology in coach education. Extending this focus on leadership, Guzmán-Rodríguez et al. show that cohesion and adaptability drive performance more than diversity alone, underscoring the need for leaders to actively nurture these qualities. Building on this gap Urgun et al.'s review cross-cultural training interventions, concluding they are effective and should be developed specifically for sports coaches. Taken together, these studies advocate for tailored, culturally responsive training and policy reform to promote inclusion in sport.

Cultural practices serve as powerful tools for inclusion and identity affirmation. Pasqua and Toledo trace Capoeira's journey using a documentary approach to show how this Afro-Brazilian polysemic practice promotes interpersonal relationships and cultural awareness. Similarly, Yang and Huang explore the preservation of Ma's Tongbei martial arts, showing how master-disciple relationships foster group cohesion that both sustains traditional techniques and enables adaptability. These findings suggest that sport education can embrace heritage-based pedagogies to promote intercultural dialogue and community engagement.

Public attitudes toward diversity in sport are shaped by complex socio-cultural dynamics. Wicker and Cunningham analyse the attitudes of 19,396 Europeans toward gender equality in sport, revealing greater support for female leadership than for media visibility of women's sports. In China, He et al. investigate how traditional gender awareness and stereotypes inhibit female participation in contact leisure sports. Chen and Ni and Wang et al. further investigate stigma in Chinese sport, focussing, respectively, on student-athletes and on specific sports, and identifying stereotype threat and social identity as key factors. Turning to issues of nationality and identity, Lu et al. examine Chinese perceptions of naturalized athletes, showing that perceived threats and benefits influence acceptance. At the global level, El-Dabt et al. critically examine sport's role in nation branding through Qatar's strategic use of mega-events, while Crossan et al. analyse the internationalization of Czech football and ice hockey sports clubs. Together, these studies reveal tensions between globalization and local identity, suggesting that soft power strategies must balance cultural authenticity with global contexts. These studies highlight the need for inclusive media representation, transparent communication, and stigma-reduction strategies.

The intersection of sport participation and culture is explored through multiple lenses. Mainra et al. highlight the role of family and friend support in promoting physical activity among Indigenous adults in Canada emphasizing the importance of community ties. Extending this theme, Xia et al. finds that social support also mediates the relationship between exercise participation and reductions in feelings of self-deficiency among Chinese university students. In a different cultural context, Collins et al. suggest that intrinsically motivated values in American student-athletes transcend demographic boundaries pointing, to a shared foundation of athletic commitment. Du and Ning compare Eastern and Western mindfulness interventions, and show that culturally adapted approaches generate stronger psychological benefits. These findings provide support for culture-sensitive strategies and community-driven interventions.

While each article presents distinct findings, collectively they uncover sources of exclusion while highlighting cultural contexts, national policies, and social support systems as key mechanisms of inclusion (cf. Wang et al., 2025). They explain the multifaceted nature of cultural diversity in sport, revealing how representation, identity, and inclusion are shaped by structural, psychological, and cultural contexts. They also demonstrate that sport is a powerful arena for negotiating belonging, heritage, and equity. Future research must continue to embrace interdisciplinary methods and participatory frameworks, ensuring that sport evolves as an inclusive domain, where diversity is recognized and embedded in practice.
